# Neuroinflammation as a Therapeutic Target for Mitigating the Long-Term Consequences of Acute Organophosphate Intoxication

**DOI:** 10.3389/fphar.2021.674325

**Published:** 2021-05-12

**Authors:** Peter M. Andrew, Pamela J. Lein

**Affiliations:** Department of Molecular Biosciences, School of Veterinary Medicine, University of California, Davis, CA, United States

**Keywords:** acquired epilepsy, astrocytes, cognitive impairment, functional polarization, microglia, glial cell activation

## Abstract

Acute intoxication with organophosphates (OPs) can cause a potentially fatal cholinergic crisis characterized by peripheral parasympathomimetic symptoms and seizures that rapidly progress to *status epilepticus* (SE). While current therapeutic countermeasures for acute OP intoxication significantly improve the chances of survival when administered promptly, they are insufficient for protecting individuals from chronic neurologic outcomes such as cognitive deficits, affective disorders, and acquired epilepsy. Neuroinflammation is posited to contribute to the pathogenesis of these long-term neurologic sequelae. In this review, we summarize what is currently known regarding the progression of neuroinflammatory responses after acute OP intoxication, drawing parallels to other models of SE. We also discuss studies in which neuroinflammation was targeted following OP-induced SE, and explain possible reasons why such therapeutic interventions have inconsistently and only partially improved long-term outcomes. Finally, we suggest future directions for the development of therapeutic strategies that target neuroinflammation to mitigate the neurologic sequelae of acute OP intoxication.

## Introduction

Organophosphates (OPs) are a group of compounds originally synthesized in the 1930s as insecticides. Throughout the 20th century, OPs were widely used to control insects in residential and agricultural settings. While their residential use has been increasingly restricted in the United States and European Union over the past several decades due to human health concerns, they remain the most widely used class of insecticides worldwide with particularly heavy use in developing countries (Voorhees et al., 2016). In the United States and most European countries, poisoning with OP pesticides is largely the result of occupational or accidental exposures; however, globally, suicidal self-poisoning with OPs annually accounts for over 200,000 deaths and significantly greater morbidity (Mew et al., 2017). Unfortunately, this lethal acute toxicity led to the weaponization of OPs as nerve agents during World War II (Costa 2018). OP nerve agents remain credible chemical threats as evidenced by their use as chemical weapons in the Syrian civil wars, and a number of high-profile terrorist attacks and assassination events, notably, the Tokyo subway and Matsumoto sarin gas attacks and the assassination of Kim Jong-Nam in Kuala Lumpur (Jett and Spriggs 2020).

Evidence from both human and animal studies establishes neurotoxicity as the primary endpoint of concern following acute intoxication with OPs. The canonical mechanism of OP neurotoxicity is inhibition of the enzyme acetylcholinesterase (Costa 2018), which normally functions to terminate cholinergic transmission throughout the central and peripheral nervous systems. Acute inhibition of acetylcholinesterase activity by > 60–80% can lead to a clinical toxidrome referred to as cholinergic crisis. This toxidrome is characterized by peripheral parasympathetic symptoms, depression of central control of respiration, seizures that can rapidly progress to *status epilepticus* (SE) and death (Eddleston et al., 2008; Hulse et al., 2014). The standard of care treatment for OP-induced cholinergic crisis, which can be fatal if not treated, includes atropine to block peripheral muscarinic receptors, oxime to reactivate acetylcholinesterase, and benzodiazepines to reduce seizure activity (Bird et al., 2003; Eddleston et al., 2008). However, if treatment is not initiated within minutes after the onset of clinical symptoms, survivors of acute OP poisoning often face significant long-term morbidity, including cognitive dysfunction, affective disorders or spontaneous recurrent seizures (SRS) that typically manifest in the weeks to months after the initial exposure (Roldan-Tapia et al., 2006; Dassanayake et al., 2007, 2008; Chen 2012; De Araujo Furtado et al., 2012; Pereira et al., 2014; Figueiredo et al., 2018). Therefore, new therapeutic strategies are needed to mitigate the long-term effects of acute OP intoxication, particularly in cases where initial treatment is delayed. However, efforts to develop more effective therapeutic approaches have been stymied by the lack of data regarding the pathogenic mechanisms underlying the chronic effects associated with acute OP intoxication.

Neuroinflammation has been proposed to be a key pathogenic mechanism underlying the chronic neurologic consequences of acute OP poisoning (Guignet and Lein 2019). Neuroinflammation refers to the immune-mediated, microglial and astrocyte-propagated responses organized within the nervous system in response to injury in the brain or systemic inflammation (Kraft and Harry 2011; Viviani et al., 2014). This hypothesis derives in part from increasing evidence over the past decade demonstrating that acute OP intoxication triggers robust neuroinflammatory responses that develop within hours and can persist for weeks to months post-exposure, preceding and/or coinciding with the onset of long-term effects. This hypothesis is further supported by clinical and experimental data implicating neuroinflammation in the pathogenesis of cognitive impairment, affective disorders and epilepsy in models other than acute OP intoxication. However, data causally linking neuroinflammation to the chronic neurologic consequences of acute OP intoxication are sparse, and the few studies that have tested therapeutic interventions that mitigate neuroinflammation in experimental models of acute OP intoxication have not consistently reported improved neurological outcomes. Here, we provide an overview of the evidence demonstrating that acute OP intoxication causes chronic neurologic outcomes, review the experimental evidence implicating neuroinflammation as a mechanism linking acute OP neurotoxicity to these long-term health effects, and discuss critical data gaps that need to be addressed to better understand the diagnostic and therapeutic implications of OP-induced neuroinflammation.

## Evidence of Chronic Neurologic Sequelae of Acute OP Intoxication

Clinical evidence suggests that survivors of acute OP intoxication may suffer long-term neurologic effects after initial effects have subsided (Chen 2012; Figueiredo et al., 2018). In a recent systematic review of the evidence for long-term effects after acute exposure to intoxicating levels of sarin nerve agent, the authors concluded that acute poisoning with sarin is known to be a neurological hazard to humans during the first 7 days following exposure and suspected to be a hazard in the weeks to years after exposure (Jett et al., 2020). Long-term effects identified in humans acutely intoxicated with sarin included reduced cholinesterase activity, visual and ocular effects, impaired learning and memory, and structural changes in the brain (Jett et al., 2020). Experimental animal models confirm that acute intoxication with sarin and other OPs is associated with chronic adverse outcomes, including persistent neuropathology, cognitive deficits and electroencephalographic abnormalities, including SRS (De Araujo Furtado et al., 2012; Pereira et al., 2014). As in humans, these neurologic sequelae develop weeks to months following recovery from the initial cholinergic crisis and are resistant to current medical countermeasures (Shih et al., 2003; Deshpande et al., 2014; Shrot et al., 2014; Kuruba et al., 2018; Wu et al., 2018; Supasai et al., 2020). Below we provide a brief review of the data demonstrating chronic effects of acute OP intoxication.

### Behavioral and Psychiatric Abnormalities Following Acute OP Intoxication

The Tokyo subway and Matsumoto sarin gas attacks in the 1990s provide the most comprehensive clinical dataset demonstrating correlations between acute OP poisoning and subsequent neurologic abnormalities. Individuals exposed to sarin during the Tokyo subway attack experienced psychomotor and memory deficits that persisted for years after the chemical attack with more highly exposed individuals exhibiting more severe cognitive deficiencies (Nishiwaki et al., 2001; Miyaki et al., 2005). Humans acutely poisoned with OP pesticides exhibit neurologic consequences similar to those observed in humans intoxicated with sarin. Some of the earliest reports of humans poisoned with OP pesticides describe memory deficits, decreased attention and alertness (Metcalf and Holmes 1969). Later studies confirmed that acute intoxication with OP pesticides was associated with impaired attention and memory as well as deficits in higher cognitive functions, such as mental abstraction (Savage et al., 1988; Rosenstock et al., 1991; Steenland et al., 1994; Stallones and Beseler 2002; Roldan-Tapia et al., 2006). Impaired performance on simple tasks like reading comprehension (Stallones and Beseler 2002), underscores the significant negative impacts of acute OP intoxication on survivors’ quality of life.

There is substantial human evidence linking repeated occupational exposures to OP pesticides and nerve agents at levels that do not cause cholinergic crisis with increased risk of psychiatric disorders, particularly depression and anxiety-related mood disorders (Levin et al., 1976; Stephens et al., 1995; Bazylewicz-Walczak et al., 1999; Mackenzie Ross et al., 2010), and with Gulf War Syndrome, a complex set of symptoms seen in Gulf War veterans that includes depression, anxiety, and memory loss (Golomb 2008). Fewer studies have evaluated the effects of acute OP intoxication on affect, but those that have indicated that acute OP poisoning also increased risk for affective disorders (Ohtani et al., 2004; Yanagisawa et al., 2006). In survivors of the Matsumoto sarin attack examined 5 years post-exposure, the incidence of psychological issues, including depressive mood, was significantly increased relative to age-matched control subjects living in the same city at the time of the attack but not exposed to sarin (Yanagisawa et al., 2006). It is challenging to dissociate the subjective experience of trauma from the effects of OP exposure on affective changes. The human data support an increased risk for post-traumatic stress disorder (PTSD) in survivors of both the Tokyo subway and Matsumoto attacks (Ohtani et al., 2004; Yanagisawa et al., 2006). However, exposed individuals displayed several psychiatric symptoms not typically associated with PTSD (Ohtani et al., 2004), suggesting a role for OP intoxication in altering affect independent of traumatic psychological experience. Consistent with this interpretation, agricultural workers acutely poisoned with OP pesticides are at a significantly increased risk for depression, anxiety and mood disorders (Stallones and Beseler 2002; Roldan-Tapia et al., 2006; Beseler et al., 2008; Wesseling et al., 2010). Moreover, a positive association has been demonstrated between the number of acute intoxication events and both the incidence of depression- and anxiety-related symptomatology and risk for suicidal ideation (Wesseling et al., 2010).

Observations from experimental animal models largely parallel the human condition. For example, rats (Filliat et al., 1999) or mice (Filliat et al., 2007) acutely intoxicated with the OP nerve agent soman exhibited marked deficits in spatial learning that correlated with neuropathology in the CA1 region of the hippocampus, a brain region integral to cognitive and mnemonic processes. While impaired cognitive function persisted at 90 days after exposure to soman, the neuropathologic effects in the hippocampus were largely resolved by this time (Filliat et al., 2007). These observations suggest that the chronic effects of acute OP intoxication on cognition and memory are not dependent on sustained neuropathologic changes in the hippocampus, implying the contribution of additional pathogenic processes. Similarly, acute intoxication with OP pesticides had long-term effects on cognitive function in animal models. In rat models of acute intoxication with diisopropylfluorophosphate (DFP), deficits in spatial learning and memory were observed within 1 month after exposure (Brewer et al., 2013), while impairments in fear conditioning manifested about 1 month post-exposure and persisted to at least 2 months post-exposure (Flannery et al., 2016; Guignet et al., 2020). Likewise, rats acutely intoxicated with paraoxon, the oxon metabolite of parathion, exhibited pronounced deficits in the novel object recognition task that persisted up to 14 weeks post-exposure (Deshpande et al., 2014).

A number of animal studies indicate that acute OP intoxication changes behaviors thought to have face validity to human affective disorders. Rats acutely intoxicated with the OP pesticide DFP (Wright et al., 2010) or paraoxon (Deshpande et al., 2014) exhibited increased immobility time in the forced swim test, a measure of despair-like depressive behavior. Paraoxon-intoxicated rats also displayed anhedonia in the sucrose preference test (Deshpande et al., 2014), another indicator of depressive-like behavior. Acute soman intoxication produced a robust and persistent anxiogenic phenotype in mice (Coubard et al., 2008) and rats (Prager et al., 2014). Similarly, rats acutely intoxicated with paraoxon exhibited increased anxiety-like behavior up to 14 weeks following exposure (Deshpande et al., 2014). Conversely, acute intoxication with DFP produced an anxiolytic effect in rats (Guignet et al., 2020). This latter observation is consistent with reports from rat models of acute sarin intoxication, in which rats demonstrated a persistent anxiolytic effect for up to 6 months post exposure (Grauer et al., 2008). While additional investigation is necessary to resolve the exact nature of the link between acute OP intoxication and affective behavior, both human and animal evidence strongly suggest that acute OP intoxication has long-term impacts on affect.

### Electroencephalographic Abnormalities in the Brain

Early studies of agricultural and industrial workers acutely intoxicated with OP pesticides or nerve agents identified subtle, yet persistent alterations in electroencephalographic (EEG) activity (Metcalf and Holmes 1969; Duffy et al., 1979). These studies, however, included individuals exposed on multiple occasions, making it difficult to determine whether a single acute intoxication event caused long-term EEG abnormalities. Nonetheless, studies of survivors of the Tokyo subway sarin attack indicated that at 1 year post-intoxication, these individuals had abnormalities in baseline EEG activity (Murata et al., 1997; Yanagisawa et al., 2006) and abnormal EEG responses to auditory and visual stimuli (Murata et al., 1997).

Experimental animal data confirm and expand these human data. SRS have been demonstrated in animals following acute intoxication with OP nerve agents or pesticides (De Araujo Furtado et al., 2010; Putra et al., 2019; Guignet et al., 2020). Rats acutely intoxicated with soman (De Araujo Furtado et al., 2010) or DFP (Guignet et al., 2020) similarly presented with a latent period of relatively normal EEG activity after the OP-induced SE that evolved into SRS within weeks following exposure. Further investigation is needed to comprehensively characterize SRS following acute OP intoxication; for example, investigators have yet to identify the factors that influence duration of the latency period, as well as the regional distribution of epileptic foci during acute OP-induced SE and during SRS. Nonetheless, parallels between the development of SRS in animal models of acute OP intoxication and the acquisition of injury-induced epilepsy in human patients suggests acute OP intoxication can trigger epileptogenic processes in human survivors.

## Neuroinflammation as a Mechanism Contributing to the Neurologic Sequelae of Acute OP Intoxication

Research to identify pathogenic mechanisms underlying the long-term neurologic consequences of acute OP intoxication have historically focused on mechanisms responsible for sustaining seizure activity. Seizure activity has been considered the primary driver of not only immediate, but also long-term neuropathology and neurologic outcomes (McDonough and Shih 1997) because of extensive experimental data demonstrating a direct correlation between seizure duration and the extent of brain damage (Lallement et al., 1994; McDonough et al., 1995; Flannery et al., 2016). Consequently, it has been hypothesized that seizure termination is an effective therapeutic strategy for mitigating the neurologic sequelae of acute OP intoxication. However, clinical (Roldan-Tapia et al., 2006; Dassanayake et al., 2007, 2008; Chen 2012; De Araujo Furtado et al., 2012; Pereira et al., 2014; Figueiredo et al., 2018) and experimental (Shih et al., 2003; Deshpande et al., 2014; Shrot et al., 2014; Kuruba et al., 2018; Wu et al., 2018; Supasai et al., 2020) data indicate that survivors of acute OP intoxication have increased risk of developing chronic neurologic effects even if they receive effective anti-seizure treatment. Additionally, a subset of rats not experiencing significant seizure activity after acute DFP intoxication at a dose that triggered sustained SE in the majority of animals had significant neuropathology, albeit the neuropathologic lesions were delayed in onset and resolved earlier than similar lesions in the majority of DFP-intoxicated animals (Gonzalez et al., 2020). Thus, while seizure severity and/or duration are significant determinants of the extent of long-term brain damage following acute OP intoxication, additional pathogenic mechanisms independent of seizure activity seem to be involved.

Neuroinflammation has emerged as a potential pathogenic mechanism contributing to the chronic neurologic consequences of acute OP intoxication. Similar to peripheral inflammatory responses, neuroinflammation is often critical to functional recovery (Yang and Zhou 2019). However, aberrant or prolonged activation of neuroinflammatory pathways is characteristic of a number of neurologic disorders, and chronic neuroinflammation is often associated with disease progression (Skaper et al., 2018; Kwon and Koh 2020). As a result, significant research effort is currently focused on understanding the role of neuroinflammation in seizures in general, and more specifically, in OP-induced SE. While a strong association between seizures and neuroinflammation has been documented in humans with seizure disorders and experimental animal models of seizures triggered by factors other than OPs (Aronica and Crino 2011; Vezzani and Viviani 2015; Rana and Musto 2018; Vezzani et al., 2019), evidence implicating neuroinflammation as a pathogenic mechanism linking acute OP intoxication to long-term neurologic consequences is more nascent.

### Neuroinflammation Following Acute OP Intoxication

An increasing body of experimental evidence indicates that acute OP intoxication triggers robust neuroinflammatory responses that evolve over the course of hours to months following exposure (Banks and Lein 2012; Guignet and Lein 2019). In rat and mouse models of acute intoxication with OP nerve agents or OP pesticides, astrocytes responded with transcriptional and translational upregulation of GFAP within hours of SE (Baille-Le Crom et al., 1995; Damodaran et al., 2002; Liu et al., 2012; Rojas et al., 2015; Rojas et al., 2018). By 24 h, GFAP immunoreactivity leveled off (Zimmer et al., 1997; Collombet et al., 2005; Collombet et al., 2007; Liu et al., 2012); however, a second increase in GFAP immunoreactivity emerged within 3–7 days and persisted up to 3 months after the initial exposure (Collombet et al., 2005; Collombet et al., 2007; Angoa-Pérez et al., 2010; Liu et al., 2012; Siso et al., 2017; Putra et al., 2019; Guignet et al., 2020).

Microglial responses to acute OP intoxication tend to manifest more slowly than astrocytic responses and differ between animal models. Following acute intoxication with OP nerve agents, microglia progressively adopted the classic ameboid morphology of activated phagocytic microglia within 2–3 days and clustered around regions of severe tissue damage (Zimmer et al., 1997; Collombet et al., 2005). In a mouse model of soman intoxication, this type of microglial response was largely diminished by 8 days post exposure (Collombet et al., 2005). In contrast, in a rat model of acute intoxication with the OP pesticide DFP, at 1 day post-exposure, ionized calcium binding adaptor molecule 1 (IBA1) was not significantly upregulated compared to vehicle control animals, and microglia were largely ramified (Flannery et al., 2016; Siso et al., 2017). However, by 2–3 days post-DFP exposure, IBA1 immunolabeling was significantly elevated and microglia had transitioned to an active ameboid morphology (Rojas et al., 2015; Flannery et al., 2016; Siso et al., 2017). IBA1 immunoreactivity continued to increase up to 7 days post-DFP exposure and elevated IBA1 immunolabeling persisted up to 2 months (Siso et al., 2017; Guignet et al., 2020). Thus, microglial responses may vary by agent (soman vs. DFP) and species (mouse vs. rat) as has been reported for other experimental situations (Colton et al., 1996; Colton 2009; Lam et al., 2017).

Acute OP intoxication also elicited pronounced shifts in the expression of inflammatory mediators. Soman upregulated transcription of the proinflammatory cytokines interleukin 1-beta (IL-1β), IL-6 and TNFα, as well as several immune cell adhesion molecules within 6 h of exposure (Svensson et al., 2001; Williams et al., 2003; Dillman et al., 2009). Some data suggested that the transcriptional upregulation persisted up to 7 days post-exposure (Dhote et al., 2007). An initial increase in protein levels of proinflammatory cytokines was seen as early as 2 h following exposure to OP nerve agents, but returned to baseline after 48 h (Chapman et al., 2006; Johnson and Kan 2010; Johnson et al., 2011). There was some evidence of a second, more enduring upregulation of proinflammatory cytokines, with one study reporting elevated levels of IL-1β, IL-6 and TNFα peptides in the rat cortex 1 month following acute intoxication with sarin (Chapman et al., 2006).

Experimental investigations of the effects of acute intoxication with OP pesticides on inflammatory mediators have yielded mixed findings. Chemokine mRNA and protein expression was largely upregulated within 1 day after DFP exposure and altered expression patterns persisted through 28 days post-exposure (Liang et al., 2018; Rojas et al., 2018; Hobson et al., 2019). However, both increased and decreased levels of pro- and anti-inflammatory cytokine mRNA and protein have been reported in rats following acute DFP intoxication (Li et al., 2015; Liang et al., 2018; Rojas et al., 2018; Hobson et al., 2019). These shifts in cytokine expression were evident within 1 day post-exposure and persisted for up to 2 weeks (Hobson et al., 2019). Additional investigation is needed to more comprehensively characterize the spatiotemporal changes in chemokines and cytokines following acute OP pesticide intoxication.

Levels of COX-2 mRNA and protein were significantly upregulated within hours of acute OP intoxication and remained elevated up to 1 week following exposure (Angoa-Pérez et al., 2010; Rojas et al., 2015; Rojas et al., 2018). PGE2 levels in the brains of rats acutely intoxicated with sarin were significantly elevated within 2 days following exposure, but returned to baseline by 6 days post-exposure (Grauer et al., 2008). A second increase in PGE2 levels was observed at 1 month following sarin intoxication that persisted for up to 6 months post-exposure (Chapman et al., 2006; Grauer et al., 2008; Chapman et al., 2015). Recently, significant shifts in the profile of other inflammatory lipids have been reported in the brain of rats following acute DFP intoxication (Yang et al., 2019). In this model, numerous proinflammatory lipid mediators were found to be upregulated in a region-specific manner between 1 and 7 days following DFP intoxication, while levels of anti-inflammatory lipids were diminished over this same time period (Yang et al., 2019).

While much of the evidence demonstrating neuroinflammatory responses triggered by acute OP intoxication comes from experimental animal models (see below), there are data suggesting a similar neuroinflammatory response may happen in humans. For example, expression of proinflammatory genes was upregulated in human astrocytes exposed to the OP pesticide chlorpyrifos *in vitro* (Mense et al., 2006). Patients with OP-associated Gulf War Illness were found to have elevated brain expression of translocator protein (TSPO), a biomarker of neuroinflammation (Guilarte 2019), by PET imaging (Alshelh et al., 2020). These studies suggest the possibility that OPs induce inflammatory pathways independent of seizure activity, as has been shown in a rat model of acute DFP intoxication (Gonzalez et al., 2020). Whether acute OP-induced SE induces neuroinflammation in humans has yet to be investigated.

## Is Neuroinflammation Causally Linked to the Long-term Neurologic Consequences Associated With Acute OP Intoxication?

As described in *Neuroinflammation as a Mechanism Contributing to the Neurologic Sequelae of Acute OP Intoxication*, there is substantial experimental evidence documenting that acute OP intoxication triggers robust neuroinflammation prior to and/or coincident with the onset of long-term neurologic effects, and increased neuroinflammation can persist for weeks to months. Whether neuroinflammation is a cause of the chronic neurologic consequences associated with acute OP intoxication remains debatable. The strongest support for this hypothesis comes from studies probing a causal role for neuroinflammation in the pathogenesis of cognitive dysfunction and epileptogenesis triggered by factors other than acute OP intoxication. For example, delayed onset of cognitive deficits and SRS are observed in experimental animal models of pilocarpine- and kainate-induced SE (Müller et al., 2009; Rattka et al., 2013), and research with these models support a contribution of neuroinflammatory processes to chronic neurologic sequelae. Similarly, a body of research ties SE-associated neuroinflammation to the development of subsequent neurobehavioral impairment. In particular, pharmacological suppression of microglial activation (Sun et al., 2015; Liu et al., 2017; Oliveira et al., 2018), modulation of cytokine levels (Somera-Molina et al., 2009; Li et al., 2017), or inhibition of mTOR signaling (Brewster et al., 2013; Schartz et al., 2016) is coincident with improved cognitive behavior across species and SE models. Additionally, there are reports that suppression of eicosanoid signaling in the context of SE attenuates cognitive deficits (Levin et al., 1976; Gobbo and O'Mara 2004). Other studies, however, suggest that COX inhibition does not attenuate SE-associated cognitive impairment (Kunz et al., 2005; Polascheck et al., 2010). It has been proposed that such conflicting data may reflect differences in pharmacological specificity for COX isoforms (Rojas et al., 2014) or that efficacy is critically tied to the timing of COX2 upregulation following SE (Jiang et al., 2015).

Across SE models, substantial evidence suggests a connection between neuroinflammation and epileptogenic processes (Rana and Musto 2018; Vezzani et al., 2019). In particular, pro-inflammatory cytokines have been causally linked to ictogenic processes *via* well-characterized molecular mechanisms (Vezzani et al., 2008; Vezzani et al., 2019). Pharmacological inhibition of COX signaling throughout experimentation attenuates the incidence, frequency, and duration of SRS following SE (Jung et al., 2006; Ma et al., 2012). However, targeted pharmacologic inhibition of COX2 during the latent period following SE provides only modest, if any, benefit in mitigating SRS across SE models (Holtman et al., 2010; Polascheck et al., 2010), suggesting that COX2 inhibition may attenuate acute seizure activity but has minimal efficacy in blocking epileptogenesis. Alternative anti-inflammatory strategies, including pharmacological inhibition of microglial activation (Wang et al., 2015), mTOR inhibition (Zeng et al., 2009), and spingosine-1-phosphate analog-mediated immunosuppression (Gao et al., 2012; Pitsch et al., 2019) have also been shown to attenuate the frequency or severity of SRS following SE.

### A Role for Neuroinflammation in the Long-Term Neurologic Effects of Acute OP Intoxication

As described in *Neuroinflammation as a Mechanism Contributing to the Neurologic Sequelae of Acute OP Intoxication*, there is substantial evidence detailing the extent and persistence of neuroinflammation following acute OP intoxication. These observations support the hypothesis that neuroinflammation may contribute to the chronic neurologic consequences of acute OP intoxication. However, only a few studies have directly tested this possibility by experimentally manipulating neuroinflammatory responses to probe their involvement in the neurologic outcomes of OP-induced SE (Table 1). The majority of studies evaluating the mechanistic role of neuroinflammation in the long-term effects of acute OP intoxication have focused on assessing the effects of blocking neuroinflammation on neuropathologic outcomes. Therapeutic strategies that inhibit prostaglandin signaling reduced histopathologic and biochemical indices of neurodegeneration and neuroinflammation in the rat brain within days of acute DFP intoxication (Rojas et al., 2015; Chapman et al., 2019). Similarly, neuregulin-1, an anti-inflammatory growth factor, reduced DFP-induced neurodegeneration and neuroinflammation when administered to rats following DFP exposure (Li et al., 2012; Li et al., 2015). Indirect targeting of inflammation *via* inhibition of oxidative stress, a process closely linked to neuroinflammation (Eastman et al., 2020), also suppressed neuroinflammation and neurodegeneration in a rat model of DFP-induced SE (Liang et al., 2018; Liang et al., 2019; Putra et al., 2019; Putra et al., 2020).

**TABLE 1:** Effects of anti-inflammatory therapeutics in OP models.

OP model	Pretreatments	Therapeutic class	Therapeutic agent	Dosing regimen	Outcome	References
Rat—Sarin (108 ug/kg, i.m.)	None	NSAID	Indomethacin Ibuprofen	10 mg/kg, at onset of convulsions	↑ seizure severity	Chapman et al. (2019)
Rat—Sarin (108 ug/kg, i.m.)	None	Steroid	Methylprednisolone Dexamethasone	20 mg/kg, at onset of convulsions	↑ seizure severity	Chapman et al. (2019)
Rat—Sarin (108 ug/kg, i.m.)	None	Steroid	Methylprednisolone Dexamethasone	20 mg/kg ip at 4, 20 hr after sarin	No improvement in clinical seizure severity or PGE2 levels at 24 and 48 h.	Chapman et al. (2019)
Rat—Sarin (108ug/kg, i.m.)	None	COX-2 Inhibitor	Nimesulide	6 mg/kg at 4, 20 h after sarin	↓ TNFa, PGE2, IL1B, and IL6 expression at 8 and 24 h;	Chapman et al. (2019)
					No improvement to neuropathology.	
Rat—Sarin (108 ug/kg, i.m.)	None	COX inhibitor	Ibuprofen	10 mg/kg at 4, 20 h after sarin	↓ IL6 expression at 24 h;	Chapman et al. (2019)
					No improvement to neuropathology.	
Rat—Sarin (108 ug/kg, i.m.)	None	Phospholipase A2 Inhibitor	Quinacrine	5 mg/kg at 4, 20 h after sarin	↓ IL1B and IL6 expression at 24 h;	Chapman et al. (2019)
					No improvement to neuropathology.	
Rat—Sarin (108 ug/kg, i.m.)	None	PGE Analogue	Ilomedin Prostin Misoprostol	Immediately after sarin, again at 2 hr after sarin	↓ TNFa, IL1B, and IL6 expression at 24 h;	Chapman et al. (2019)
					Prostin and misoprostol ↓ TSPO expression at 24 h;	
					At 7 d, all-or-none reduction in TSPO.	
Rat—Soman (154 ug/kg, s.c.)	30 m asoxime chloride (125 mg/kg, i.m.)	Antioxidant	AEOL10150	7 mg/kg, s.c. 1, 5, or 15 min after SE onset, repeated every 4 h	↓ oxidative stress, microglial activation, neurodegeneration, and proinflammatory cytokine expression at 24 h.	Liang et al., (2019)
Rat—Soman (180 ug/kg, s.c.)	30 m asoxime chloride (125 mg/kg, i.m.)	Immune modulator	Poly-YE	1 mg/kg, s.c. 24 h post intoxication	↓ neurodegeneration and microglial activation in the piriform cortex at 28 d;	Finkelstein et al. (2012)
					No significant improvements in Barnes Maze performance.	
Rat—DFP (9.5 mg/kg, i.p.)	30 m with pyridostigmine bromide (0.1 mg/kg, s.c.), 10 m with atropine methylbromide (20 mg/kg, s.c.)	EP2 receptor antagonist	TG6-10–1	5 mg/kg, i.p.	Treatment iii) ↓ delayed mortality, ↑ weight gain, ↓ transcription of cytokines/chemokines, ↓ IBA1 mRNA and immunolabeling, ↓ FJB in CA1 at 4 d.	Rojas et al. (2015)
				i) 1 h prior to DFP;		
				ii) two injections (4 and 21 h after SE-onset);		
				iii) six injections (80–150 min, 5–6 h, 9–21 h, 31–42 h, and 48 hr after SE-onset)		
Rat—DFP (9.5 mg/kg, i.p.)	30 m with pyridostigmine bromide (0.1 mg/kg, s.c.), 10 m with atropine methylbromide (20 mg/kg, s.c.)	EP2 receptor antagonist	TG6-10–1	5 mg/kg, i.p.	Treatment iii) improved discrimination in the NOR at 4 w post intoxication.	Rojas et al. (2016)
				i) 1 h prior to DFP;		
				ii) three injections (1.5, 6, 21 h after SE-onset);		
				iii) six injections (1.5, 6, 21, 30, 45–47, and 52–55 h after SE-onset)		
Rat—DFP (4 mg/kg, s.c.)	None	iNOS inhibitor	1400 W	20 mg/kg every 12 h for 3 d	↓ GFAP and IBA1 cells at 7 d;	Putra et al. (2019)
					↓ incidence, duration, and frequency of SRS over 12 w;	
					↓ reactive astrogliosis and microgliosis at 12 w.	
Rat—DFP (4.5 mg/kg, s.c.)	30 m pyridostigmine (0.1 mg/kg, i.m.)	Antioxidant	AEOL10150	5 mg/kg s.c. 5 min into SE, repeated every 4 h	↓ proinflammatory mediators at 24 h.	Liang et al. (2018)
Rat—DFP (5 mg/kg, i.p.)	None	Immune modulator	Naltrexone	5 mg/kg starting 1 h post intoxication, repeated daily.	Reduced learning deficits over 4 w post intoxication.	Brewer et al. (2013)
Rat—DFP (9 mg/kg, i.p.)	30 m pyridostigmine bromide (0.1 mg/kg, i.m.), 10 m atropine methylnitrate (20 mg/kg, i.m.)	Growth factor	Neuregulin-1 (NRG-1)	3.2 ug/kg, internal carotid artery	Treatment i) at 24 h ↓ neurodegeneration and oxidative stress; ↓ shifts in microglia activation; ↓ expression of proinflammatory cytokines;	Li et al. (2012); Li et al. (2015)
				i) 5 min pretreatment;	Treatment ii) ↓ neurodegeneration at 24 h.	
				ii) post-treatment 1 hr after DFP intoxication;		
				iii) post-treatment 4 h after DFP intoxication		
Rat—DFP (4 mg/kg, s.c.)	None	Antioxidant	Diapocynin (DPO)	300 mg/kg, p.o., six doses, 12 h intervals beginning 2 h post-DFP	Mitigates motor deficits at 18 d;	Putra et al. (2020)
					↓ astrogliosis, neurodegeneration, and inflammatory cytokine expression at 6 w.	
Rat—Paraoxon (0.45 mg/kg, i.m.)	None	Immune modulator	Poly-YE	1 mg/kg, s.c. 24 h post intoxication	↓ neurodegeneration in the piriform cortex and amygdala at 28 d;	Finkelstein et al. (2012)
					↓ microglial activation and ↑ BDNF in the piriform cortex at 28 d.	

DFP, diisopropylfluorophosphate; TMB4, trimedoxime bromide; MDZ, midazolam; AMN, atropine methyl nitrate; AS, atropine sulfate; 2-PAM, pralidoxime; SE, status epilepticus; ↑, increase; ↓, decrease; FJB, Fluoro-Jade B; NOR, novel object recognition.

Few studies have evaluated the role of neuroinflammation in the development of long-term neurologic outcomes associated with acute OP intoxication, and those that have provide conflicting results. Pharmacologic antagonism of the PGE2 receptor (EP2) following DFP-induced SE significantly reduced neuroinflammation and neurodegeneration in the rat brain (Rojas et al., 2015). This treatment also attenuated chronic deficits in learning and memory tasks but did not mitigate OP-associated changes in anxiety-like behavior or delayed mortality (Rojas et al., 2016). Similarly, reduced production of reactive oxygen species (ROS) *via* inhibition of inducible nitric oxide synthase (iNOS) mitigated DFP-induced neuroinflammation, evidenced as significantly decreased activation of microglia and astrocytes, and decreased expression of proinflammatory cytokines coincident with a lower incidence, frequency, and intensity of SRS (Putra et al., 2019). However, reduced neuroinflammation does not always coincide with functional improvements. For example, enhancing regulatory T cell activity following acute soman or paraoxon intoxication reduced microglial activation and neurodegeneration at 28 days post-exposure in the rat amygdala and piriform cortex, but did not improve performance on the Barnes maze (Finkelstein et al., 2012). Administration of the antioxidant diapocynin following acute DFP intoxication lowered proinflammatory cytokine expression and astrogliosis, but did not mitigate learning and memory deficits in the Morris water maze in rats acutely intoxicated with DFP (Putra et al., 2020).

There is indirect evidence of a causal association between neuroinflammation and functional deficits downstream of OP-induced SE. Rats administered the anesthetic urethane following acute DFP intoxication had greater reduction in neuroinflammation and development of SRS compared to rats given diazepam after DFP (Rojas et al., 2018). Urethane also provided more complete seizure control than diazepam, making it is impossible to determine the extent to which reduced neuroinflammation is responsible for SRS suppression (Rojas et al., 2018). Similarly, daily administration of naltrexone, an opioid receptor antagonist with anti-inflammatory properties (Younger et al., 2014), following DFP-induced SE improved performance of DFP intoxicated rats in the Morris water maze (Brewer et al., 2013). However, whether decreased inflammation was responsible for the improved cognition is not known because inflammation was not directly measured (Brewer et al., 2013). While these studies support the hypothesis that neuroinflammation is associated with functional deficits following OP-induced SE, they do not confirm a causal relationship.

While it has yet to be definitively demonstrated that neuroinflammation mediates the long-term neurologic effects of acute OP intoxication, current evidence suggests neuroinflammation likely contributes to at least a subset of these neurologic sequelae. Additional research efforts are needed to directly investigate the role of neuroinflammation in OP-associated outcomes. The OP research community can look to analogous studies in non-OP SE models for guidance.

## The Complexities of Neuroinflammation as a Therapeutic Target

The complex relationship between neuroinflammation and the development of long-term outcomes following SE continues to stymie therapeutic advancement. There are sufficient clinical data demonstrating that inflammatory processes exacerbate or perpetuate established epilepsy. Indeed, suppression of inflammatory pathways is efficacious in the treatment of some refractory epilepsies (Vezzani and Granata 2005). Yet the role of neuroinflammation during acute SE and the latent epileptogenic period remains ambiguous. As described above, extensive data depicts both neuroprotective and neurotoxic roles of the neuroinflammatory response, but the relative contributions of these different responses to functional outcomes of SRS and cognitive/behavioral impairment remain obscure. However, evaluation of the available data suggests several possible explanations for these seemingly discrepant findings.

### The Heterogeneous Functional Nature of the Neuroinflammatory Response

The neuroinflammatory response involves the coordinated function of numerous cellular mediators and soluble molecules. To date, SE research has generally taken a blunt approach to targeting inflammatory processes by providing therapeutics with broad anti-inflammatory activity. While there are instances in which such strategies provide therapeutic benefit (Ma et al., 2012), it may be advantageous to use more nuanced approaches. For example, COX inhibitors target a key enzyme in prostaglandin synthesis to alter the production of many different eicosanoids, which have differing effects on recovery following brain insult (Lopez-Vales and David 2019). COX inhibition decreases synthesis of PGE2, which is associated with pro-ictogenic activity in pentylenetetrazole (PTZ)-induced seizures in rats (Oliveira et al., 2008) and neurodegeneration in a mouse model of pilocarpine-induced SE (Jiang et al., 2012). However, COX inhibition also decreases synthesis of PF2α, which exerts antiseizure effects in a mouse model of kainate-induced SE (Kim et al., 2008), and PGD2, which attenuates PTZ-induced seizures in mice (Kaushik et al., 2014). Thus, non-specific inhibition of COX activity impacts prostaglandin signaling associated with both protective and deleterious effects following chemical-induced SE. Inhibition of these functionally divergent signaling pathways could account for some of the seemingly disparate effects of COX inhibition on neurological outcomes following SE. More directed treatments, such as those selectively targeting PGE2 signaling, have more consistently produced beneficial effects on outcomes following SE (Jiang et al., 2012; Rojas et al., 2015; Rojas et al., 2016).

**FIGURE 1:**
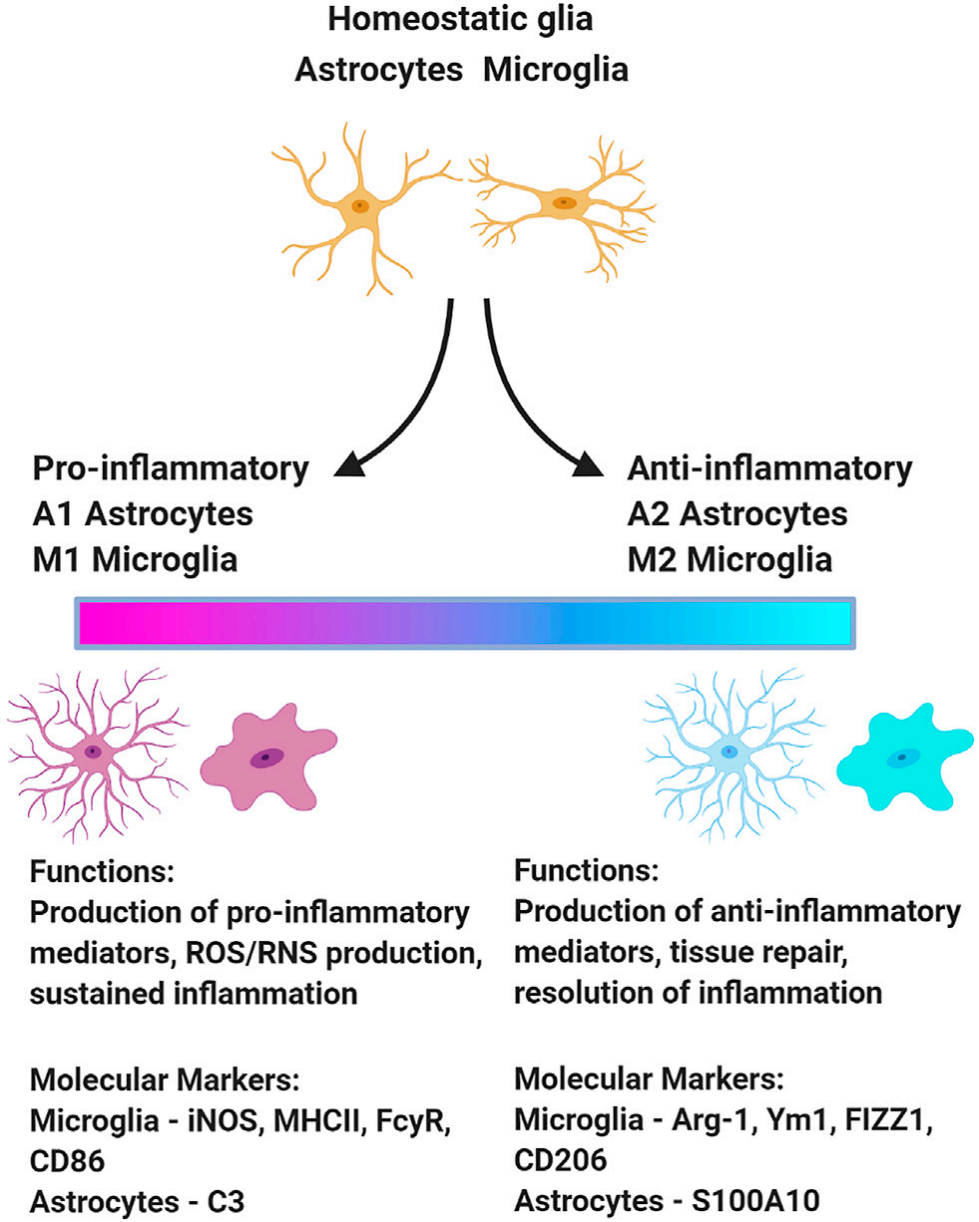
Upon insult, microglia and astrocytes can adopt a variety of phenotypes associated with different functions. This schematic presents one model of the functional polarization of microglia and astrocytes following acute OP intoxication illustrating a continuum from the extreme pro-inflammatory to anti-inflammatory state. There is some evidence of phenotypic diversity of microglia and astrocytes in animal models of acute OP intoxication (Maupu et al., 2021). Figure created with BioRender.com.

Similarly, neuroinflammation encompasses heterogeneous activational states of microglia and astrocytes. Many investigations that measure microglia and astrocyte activation as biomarkers of neuroinflammation equate astrocyte and microglia hypertrophy with neurotoxic outcomes; however, the morphology of microglia and astrocytes is not a reliable indicator of functional status. Activation-associated changes to their morphology have been linked to diverse functional phenotypes (Figure 1), including both neurotoxic and neuroprotective activities (Olah et al., 2011; Liddelow and Barres 2017; Adams and Gallo 2018). As the field moves forward, it will be critical to consider the functional heterogeneity among microglia and astrocytes exhibiting morphologies classically associated with activation.

Single-cell transcriptomic approaches can be effectively employed to identify functional subsets of microglia and myeloid cells in the CNS *via* their unique transcriptional profiles (Liddelow et al., 2017; Jordão et al., 2019; Masuda et al., 2019). Microglial subtypes are induced in a context-dependent manner with distinct transcriptional footprints associated with specific CNS conditions, including homeostasis vs. brain damage or disease (Jordão et al., 2019; Masuda et al., 2019). Similarly, subtypes of reactive astrocytes are recognized across various homeostatic and disease states (Pestana et al., 2020). The functional polarization of microglia and astrocytes (Figure 1) has not been comprehensively investigated in experimental models of acute OP intoxication. The debate surrounding the M1/M2 and A1/A2 paradigm must be acknowledged (Ransohoff 2016; Escartin et al., 2021), but characterizing the functional polarization of microglia and astrocytes following OP-induced SE would provide critical insight regarding the role of microglia and astrocytes in mediating the neurologic sequelae of acute OP intoxication. This in turn would inform therapeutic strategies to preserve the neuroprotective actions of microglia and astrocytes while blocking their injurious effects.

### Temporal Changes in the Neuroinflammatory Response

The inconsistent therapeutic efficacy of approaches that block neuroinflammation following OP-induced SE may be due to the neuroinflammatory response changing with time post-exposure. The role of inflammatory mediators following chemical-induced SE varies across the phases of acute SE, the latent period of epileptogenesis, and chronic epilepsy. In experimental animal models of chemical-induced SE, administration of anti-inflammatory therapeutics prior to SE induction exacerbated both acute seizure activity and subsequent neuroinflammation and did not decrease neurodegeneration (Gobbo and O'Mara 2004; Takemiya et al., 2006; Koo et al., 2018; Chapman et al., 2019). Conversely, supplementation with proinflammatory PGE2 analogs at the time of sarin exposure attenuated neuroinflammation triggered by SE (Chapman et al., 2019). Such data suggest that during the very acute phases of SE, neuroinflammatory processes decrease seizure severity and subsequent neuroinflammation. Delayed administration of a COX2 inhibitor several hours after SE decreased the neuroinflammatory response to acute sarin intoxication (Chapman et al., 2019) and attenuated neurodegeneration following kainate-induced SE (Gobbo and O'Mara 2004; Takemiya et al., 2006). These observations indicate that timing of pharmacologic inhibition of neuroinflammation is critically important and the therapeutic window likely varies depending on the therapeutic target and the model of chemical-induced SE.

Recent studies also hint to a similar impact of time post-SE with regards to the role of microglia. The depletion of microglia prior to pilocarpine- or kainate-induced SE exacerbated acute seizure activity (Liu et al., 2020; Wu et al., 2020), demonstrating that microglia serve a critical protective role during the acute phase of chemical-induced SE, and may reflect microglial regulation of excitatory neurotransmission during SE (Liu et al., 2020). The neuroprotective role of microglia during acute injury is also observed in other models of CNS insult not related to seizures (Rice et al., 2015; Szalay et al., 2016; Jin et al., 2017), suggesting involvement of acute protection beyond regulation of excitatory neurotransmission.

The role of microglia with increasing time after chemical-induced SE is largely undefined. Only one investigation thus far has evaluated the effects of microglial depletion post-SE on the development of SRS (Wu et al., 2020). Microglial depletion beginning 20 days after kainate-induced SE aggravated the frequency, intensity, and duration of SRS in mice (Wu et al., 2020). While the authors report that SRS developed within 28 days post-exposure in their mouse model of kainate-induced SE, they did not evaluate the time course of SRS development. Thus, it is possible that exacerbation of chronic SRS could reflect the absence of microglia and their protective function during acute SRS events. In other words, if microglia are depleted at a time when animals have already developed SRS, then microglia depletion may aggravate seizure activity as it does during the acute SE phase. In order to resolve this question, further studies are required in which microglia are depleted during the latent epileptogenic phase. The relevance of this approach is suggested by studies demonstrating that the delayed depletion of microglia in other neuroinflammatory models hastened functional recovery and promoted resolution of neuroinflammation (Rice et al., 2015; Rice et al., 2017; Lloyd et al., 2019).

While less frequently considered, a similar role has been proposed for astrocytes following SE in a rat model of acute sarin intoxication (Lazar et al., 2016). Experimental findings suggest that initial astrocytic responses are neuroprotective, whereas a second “wave” of activation in the first few days post-SE promotes acute neurodegeneration (Lazar et al., 2016). Consistent with these observations, a recent functional characterization of astrocytic responses in a mouse model of acute DFP intoxication suggested that neurotoxic “A1” astrocytes emerge several days post-SE (Maupu et al., 2021). Specific functional subsets of astrocytes have not been targeted by therapeutic intervention; therefore, the relative contributions of A1 vs. A2 astrocytes to the neurologic sequelae of SE induced by OPs or other chemicals remain in question.

### Proposed Model of the Temporal Changes in the Functional Role of Neuroinflammation Following Acute OP Intoxication

The neuroprotective vs. neurotoxic roles of neuroinflammation may continue to evolve in the weeks and months following acute OP intoxication (Collombet 2011; Banks and Lein 2012). Collombet posits that astrocyte-derived neurotrophic factors released during a late wave of astrocyte activation support neuronal cell survival and promote proliferation of neural progenitors (Collombet 2011). The proposed “beneficial” role of delayed astrocyte activation derives from two experimental observations: 1) astrocytes secreted a number of factors with well-defined neuroprotective and neurogenic functions (e.g., BDNF, VEGF); and 2) delayed neurodegeneration following soman-induced SE correlated temporally with diminished astrocyte activation. The Collombet model, however, is based upon data from a mouse model of acute soman intoxication in which there was minimal microglial activation in the weeks following acute OP intoxication (Collombet et al., 2005). More recent data from rat models of acute DFP intoxication showed rapid activation of both microglia and astrocytes that persisted for weeks following the initial insult (Flannery et al., 2016; Siso et al., 2017; Putra et al., 2019; Guignet et al., 2020). Together, these observations suggest that the spatiotemporal profile of neuroinflammation likely varies depending on the context in which SE is triggered.

As discussed above, there is evidence showing that the functional role of microglia shifts over the course of the neuroinflammatory response following acute brain injury in a variety of neurologic diseases not related to seizures (Rice et al., 2015; Szalay et al., 2016; Jin et al., 2017; Rice et al., 2017; Lloyd et al., 2019). These data suggest that the functional profile of microglia similarly changes with time following acute OP intoxication. Consistent with this possibility, temporal shifts in microglial phenotype are observed in non-OP models of SE (Benson et al., 2015), and evidence from a mouse model of acute DFP intoxication hints at similar changes in microglial phenotype during the first several days following DFP-induced SE (Maupu et al., 2021). More research is needed to fully characterize the spatiotemporal profile of microglial polarization over more extended periods of time after acute OP intoxication and to determine the effects of interventions targeting functional subsets of microglia on long-term neurologic consequences.

Microglia are known to regulate astrocytic polarization (Liddelow et al., 2017), suggesting the neuroprotective vs. neurotoxic potential of astrocytes may be related to changes in microglia polarization (Liddelow and Barres 2017). Thus, in situations with robust and potentially shifting profiles of microglial activation, it is likely that the functional polarization of microglia critically influences the functional polarization of astrocytes, and ultimately, the general direction of the neuroinflammatory response following acute OP intoxication. Indeed, there is evidence that neurotoxic C3-positive astrocyte polarization develops by 3 days after kainate- or DFP-induced SE (Wei et al., 2020; Maupu et al., 2021) and remains elevated at 6 weeks post intoxication (Putra et al., 2020). Importantly, this astrocytic polarization was dependent on microglial function (Wei et al., 2020), suggesting that microglia promoted the delayed development of neurotoxic astrocytes following acute OP intoxication.

**FIGURE 2:**
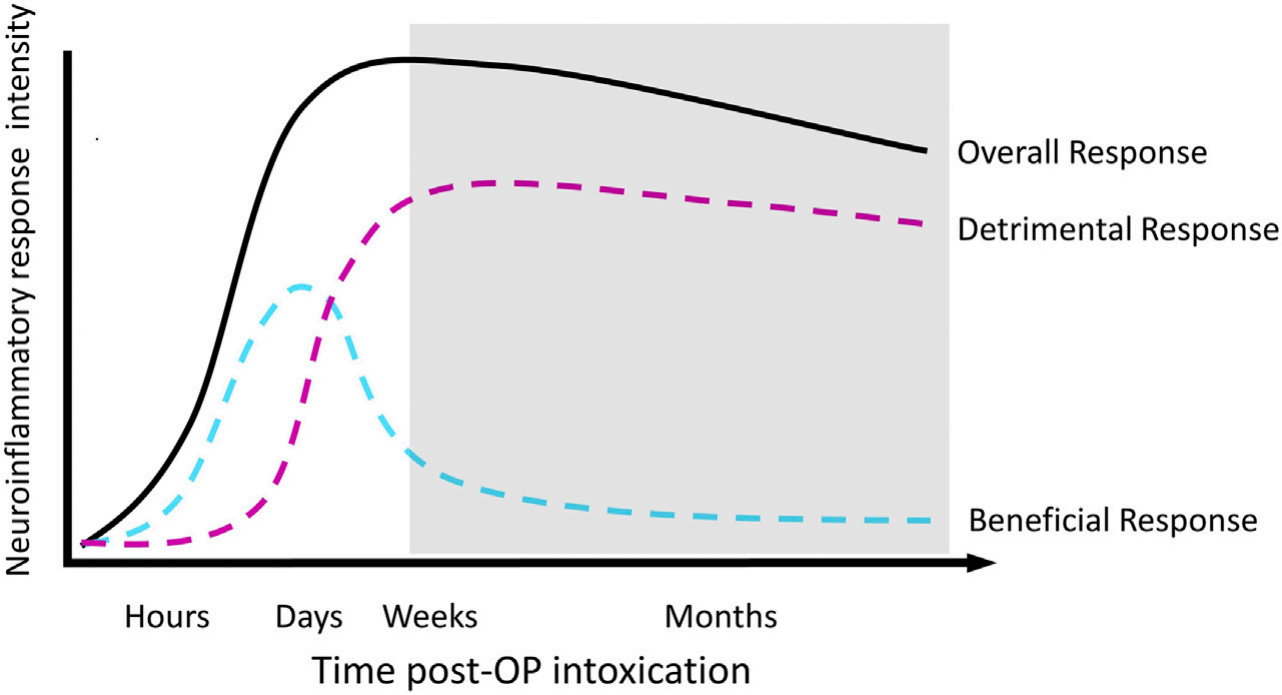
Proposed temporal profile of the neuroinflammatory response to acute OP intoxication. The shaded area depicts the development of chronic neurological sequelae.

We propose a model of the relationship between neuroinflammation and functional outcomes over time following OP-induced SE (Figure 2) in which acute neuroinflammatory responses are beneficial, while sustained neuroinflammatory responses are detrimental (Yang and Zhou 2019). The model illustrated in Figure 2 divides the neuroinflammatory response broadly into “beneficial” and “detrimental” responses; however, the composition of each component undoubtedly shifts over time. For example, the classical microglial response may be beneficial in the short-term but detrimental in chronic situations. Regardless, the overall trend remains the same; a response that is initially beneficial becomes detrimental over time. As an intentional simplification, this design does not distinguish between the individual contributions of the diverse mediators involved in the neuroinflammatory response, leaving room for the incorporation of discrete cell populations or inflammatory processes into the classifications of “beneficial” or “detrimental.” The currently available therapeutic data are generally consistent with this model. Thus, administration of an anti-inflammatory treatment prior to, during or shortly after SE is most frequently associated with more severe or longer seizure activity and/or more extensive neuropathology (Baik et al., 1999; Kunz and Oliw 2001; Holtman et al., 2010; Duffy et al., 2014; Radu et al., 2017; Chapman et al., 2019). Conversely, delayed administration of an anti-inflammatory during the hours and/or days following termination of SE tended to more reliably improve long-term outcomes (Kunz et al., 2005; Jiang et al., 2013; Jiang et al., 2015; Radu et al., 2017).

While rigorous experimentation is needed to validate our proposed model of the functional role of neuroinflammation following acute OP intoxication, it advocates for the evaluation of current anti-inflammatory therapeutics at more delayed times after SE induced by acute OP intoxication and other triggers. It should be noted that this proposed scheme is compatible with the Collombet framework (Collombet 2011) in that astrogliosis in the absence of robust and persistent “M1” microglial responses could be beneficial. However, emerging data suggests that persistent microglial activation contributes to adverse astrocytic activation and ultimately a detrimental neuroinflammatory response. It is likely that among the multiplicity of actors involved in the SE-induced neuroinflammatory response, many have time-dependent neuroprotective vs. neurotoxic roles. To better understand the functional role of neuroinflammation following acute OP intoxication, it is critical that the field more accurately defines the time-dependent functional profile of independent neuroinflammatory components. In the near future, these studies will need to be conducted using animal models; however, with increasing interest in developing radioligands for positron emission tomography (PET) that selectively recognize functional subsets of microglia and astrocytes, it may be possible to translate preclinical data to the clinical setting.

## Conclusion

Research on acute OP intoxication has made significant advances in characterizing the long-term effects of OP-induced SE. Despite these advances, limited progress has been made in developing therapeutics that meaningfully improve long-term neurologic effects. Neuroinflammation is emerging as a potentially critical determinant of SE-associated neurologic consequences. Substantial evidence indicates that the spatiotemporal profile of neuroinflammation is consistent with a proposed role in the various neurologic sequelae linked to acute OP intoxication; however, therapeutic interventions targeting neuroinflammation in experimental animal models of chemical-induced SE have been equivocal. This likely reflects differences in the timing of therapeutic interventions relative to shifts in the functional profile of microglia and astrocytes as a function of time post-exposure. Increased understanding of the spatiotemporal profiles of functional polarization of microglia and astrocytes coupled with increasing availability of biomarkers for distinguishing their different functional states will enable more targeted therapeutic strategies and optimal therapeutic windows that preserve the protective functions and attenuate the neurotoxic effects of microglia and astrocytes.
